# Removal of the Mitochondrial Fission Factor Mff Exacerbates Neuronal Loss and Neurological Phenotypes in a Huntington's Disease Mouse Model

**DOI:** 10.1371/currents.hd.a4e15b80c4915c828d39754942c6631f

**Published:** 2018-07-26

**Authors:** Moon Yong Cha, Hsiuchen Chen, David Chan

**Affiliations:** California Institute of Technology; California Institute of Technology; California Institute of Technology

## Abstract

Objective: Excessive mitochondrial fission has been associated with several neurodegenerative diseases, including Huntington’s disease (HD). Consequently, mitochondrial dynamics has been suggested to be a promising therapeutic target for Huntington’s disease. Mitochondrial fission depends on recruitment of Drp1 to mitochondria, and Mff (mitochondrial fission factor) is one of the key adaptor proteins for this process. Removal of Mff therefore greatly reduces mitochondrial fission. Here we investigate whether removal of Mff can mitigate HD-associated pathologies in HD transgenic mice (R6/2) expressing mutant Htt.

Method: We compared the phenotype of HD mice with and without Mff. The mice were monitored for lifespan, neurological phenotypes, Htt aggregate formation, and brain histology.

Results: We found that HD mice lacking Mff display more severe neurological phenotypes and have shortened lifespans. Loss of Mff does not affect mutant Htt aggregation, but it accelerates HD pathology, including neuronal loss and neuroinflammation.

Conclusions: Our data indicate a protective role for mitochondrial fission in HD and suggest that more studies are needed before manipulation of mitochondrial dynamics can be applied to HD therapy.

## INTRODUCTION

Huntington's disease (HD) is an autosomal dominant, neurodegenerative disease characterized by progressive, abnormal involuntary movements (chorea), rigidity, cognitive decline, and psychiatric symptoms^1^. There is marked loss of neurons in the caudate nucleus, putamen, and cerebral cortex^2^^,^^3^. The disease is caused by a CAG triplet expansion in exon 1 of the *HTT* (*huntingtin*) gene^4^. This mutation results in an enlarged stretch of polyglutamines in the N-terminus of Htt, with the length correlating with severity of disease. Disease alleles containing 40 or more CAG repeats are fully penetrant^1^^,^^5^. There is evidence that Htt with an expanded polyglutamine region impairs neuronal function via a toxic gain-of-function effect, in part because polyglutamine repeats are prone to aggregation. Mutant Htt has been shown to interact with multiple proteins and to interfere with both cytoplasmic and nuclear functions^6^. Mutant Htt associates with mitochondria^7^, and this organelle is among the potential cellular targets of mutant Htt. HD mutant cells have been shown to have defective mitochondrial function, including ATP production^8^, calcium handling^8^^,^^9^, transport^7^^,^^10^^,^^11^ and dynamics^10^^,^^11^^,^^12^^,^^13^.

Mitochondria are dynamic organelles whose functions are dependent on appropriate balancing of fusion versus fission^14^^,^^15^. Mitochondrial fission is mediated by Drp1 (dynamin related protein 1), a large GTP hydrolyzing enzyme of the dynamin superfamily. During mitochondrial fission, Drp1 is recruited from the cytosol onto the mitochondrial surface by one of several outer membrane proteins that serve as Drp1 receptors. There are currently four putative Drp1 receptors--Fis1, Mff, MiD49, and MiD51^14^. Although Fis1 clearly functions to recruit the Drp1 ortholog, Dnm1p, in yeast, its role in mammalian cells is currently unclear. Cells lacking Fis1 show little or no defect in mitochondrial fission^16^^,^^17^. Mff has a prominent role in recruiting Drp1, and cells lacking Mff show elongated mitochondrial tubules and have substantially less Drp1 on mitochondria^16^^,^^17^. MiD49 and MiD51 also recruit Drp1, but the recruited Drp1 appears to be kept, at least initially, in an inactive state^17^^,^^18^.

Expression of mutant Htt appears to result in aberrantly increased mitochondrial fission. HD patient cells, as well as cells engineered to express mutant Htt, show mitochondrial fragmentation due to activation of Drp1^10^^,^^11^. Mutant Htt physically interacts with Drp1 and elevates its GTP hydrolysis activity^10^^,^^11^. Two studies suggest that inhibiting mitochondrial fission has therapeutic effects in HD cell and animal models. First, in cultured striatal neurons expressing mutant Htt, treatment with the Drp1 inhibitor Mdivi1 (mitochondrial division inhibitor 1) improved mitochondrial morphology, reduced reactive oxygen species (ROS), and improved cell viability^19^. A recent report, however, questions the specificity of Mdivi1 by showing that it has effects on mitochondrial respiration and ROS production unrelated to its activity against Drp1^20^. Second, treatment of cell and mouse models of HD with P110, a peptide inhibitor of Drp1, restored normal mitochondrial morphology, improved mouse behavioral deficits, and prolonged lifespan^21^. P110 was designed to block the interaction of Drp1 with Fis1^22^. As noted above, Fis1 is a mitochondrial outer membrane protein postulated to recruit Drp1 from the cytosol onto the mitochondrial surface. These findings have raised the intriguing possibility that mitochondrial fission is an attractive therapeutic target for HD patients.

Given these results showing the functional importance of Drp1 in HD pathogenesis, we tested whether removal of Mff could ameliorate the neurological phenotypes found in the *HD*^R6/2^ mouse model. Surprisingly, we find that removal of Mff worsened the neurological phenotypes of *HD^R6/2^*mice. Although loss of Mff did not increase the number of Htt-positive aggregates, it was associated with increased neuronal loss, astrogliosis, and neuroinflammation.

## METHODS

**Transgenic mice**

Female mice with ovaries transplanted from *HD^R6/2^* mice were obtained from The Jackson Laboratory (Bar Harbor). *Mff^gt/gt^* mice lack all Mff isoforms, and their generation has been described^15^ . Ovarian transplanted (OT) *HD^R6/2^* females were crossed with *Mff^gt/gt^* males to generate *Mff^+/-^*, *HD^R6/2^* males. *Mff^gt/+^* females were crossed with *Mff^gt/+^*, *HD^R6/2^* males to generate the following littermate cohorts: *Mff^+/+^*;*Mff^+/+^*, *HD^R6/2^*;*Mff^gt/gt^; Mff*^*gt/gt*^,* HD^R6/2^*. Both the *Mff^gt/gt^* and *HD^R6/2^* lines are on mixed genetic backgrounds. The CAG repeat numbers in the relevant cohorts were determined by genomic DNA analysis by Laragen (Culver City, CA). The average CAG repeat number did not vary significantly between the *HD*^*R6/2*^ and *Mff^*g*t/gt^*, *HD*^*R6/2*^ cohorts and are noted in the figure legends.

Four cohorts of 15 animals were used. This study was approved by the Caltech Institutional Animal Care and Use Committee, and mouse maintenance and experiments were conducted in accordance with approved protocols. Humane endpoints were established and included >15% weight loss, >10% dehydration, pain, distress, or inability to ambulate. None of the experimental animals met these criteria. Cohorts were sacrificed by CO_2_ inhalation at 12 weeks for histological and biochemical analysis.

## Behavioral analysis and sample preparation

Body weight measurement and clasping assessment were evaluated weekly from 6-11 weeks of age. The clasping assessment test was performed by suspending mice by the tail for 30 s and then recording hindlimb clasping behavior. Grip strength measurement and the open field test were evaluated at 10 weeks of age. For the grip strength test, mice were placed towards the pull bar (Chatillon grip strength meter, Columbus instruments), and forelimb grip forces were measured until they released their grip from the bar. For the Open Field test, mice were allowed to move around the chamber freely. Total travelled distance was recorded with a digital camera using EthoVision software (Noldus).For biochemical analysis, mice were anesthetized with isoflurane, sacrificed, and transcardially perfused with ice-cold PBS. The striatum was microdissected from the right hemispheres and stored at -80°C until Western blot analysis. Left hemispheres were post-fixed with formalin (Sigma-Aldrich) and processed for immunohistochemistry.

**Immunohistochemistry**

Serial 30 μM coronal brain tissue sections were cut with a cryowmicrotome (Microm HM550, Thermo Scientific). For visualization of target molecules, brain tissue sections were immunostained with the following primary antibodies: EM48 (1:1000; Millipore), anti-NeuN (1:1000; Millipore), anti-glial fibrillary acidic protein (GFAP; 1:1000, Sigma-Aldrich), anti-ionized calcium binding adaptor molecule-1 (Iba-1; 1:500, Wako). Fluorescent conjugated secondary antibodies were obtained from Thermo Fisher: goat-anti-mouse Alexa 488 (1:500) and goat-anti-rabbit Alexa 568 (1:500). All stained sections were mounted on micro slides (VWR) with Fluro-Gel (EMS). For Nissl staining, tissue sections were washed with PBS and mounted on micro slides (VWR). Slides were dried at room temperature for overnight and stained with cresyl violet (Sigma-Aldrich) for 3 min. Stained sections were cover-slipped in micro slides (VWR) with xylene-based mounting medium.

**Western blotting**

Mouse brains were lysed in 1% Triton X-100 buffer (10 mM Tris, pH 7.4, 1% Triton X-100, 150 mM NaCl, 10% glycerol, and 0.2 mM PMSF) containing protease inhibitors (Sigma-Aldrich). After centrifugation at 15,000 x gfor 20 min at 4 °C, the supernatant was collected as the Triton-soluble fraction. The Triton-insoluble pellet was resuspended in lysis buffer containing 10 mM Tris (pH 7.4), 4% SDS buffer. Protein concentrations were determined with the DCTMprotein assay kit (Bio-Rad). Protein samples were separated on NuPAGE 3–8% Tris-Acetate gels (Thermo Fisher) and transferred to a PVDF membrane. Membranes were incubated with the following primary antibodies: EM48 (1:1000; Millipore), anti-beta-actin (1:10000, Sigma-Aldrich). Immunoreactivity was visualized by a chemiluminescent HRP substrate (Millipore).

**Quantification of Immunoreactivity**

For quantification of immunoreactivity, tissue sections were obtained from the striatum. Five random acquisition areas in the striatum were considered for each tissue section. NeuN-positive or Nissl-positive neurons were counted using ImageJ software (National Institutes of Health). To quantify the GFAP or Iba-1-positive areas, the immunofluorescence region in the striatum was analyzed using the ImageJ software (National Institutes of Health).

**Statistical analysis**

Statistical significance of data was analyzed with ANOVA test by Prism 6 software (GraphPad). Results are presented as means ± standard error of the mean. Survival of different cohorts were analyzed by Kaplan-Meier survival and log-rank analysis.

## RESULTS

**Removal of Mff exacerbates behavioral phenotypes in the mouse *HD^R6/2^* model**

In addition to weight loss, the mouse *HD^R6/2^* model has been documented to have several features of neurological disease, including limb clasping behavior, reduced forelimb grip strength, and diminished spontaneous motor activity. To address the effect of Mff on HD pathology, we designed a mating scheme to generate *HD^R6/2^* mice lacking *Mff*. In a previous study^15^, we engineered a mouse line (*Mff^gt/gt^*) containing a gene trap insertion within the *Mff* locus that constitutively eliminates expression of all Mff protein isoforms, results in secondary reduction of Drp1 levels, and causes a severe mitochondrial fission defect. We first crossed *Mff^gt/gt^* mice with ovarian transplanted females that were hemizygous for *HD^R6/2^* to generate *Mff^gt/+^*, *HD^R6/2^* males. These males were crossed with *Mff^gt/+^* females to generate experimental (*Mff^gt/gt^*, *HD^R6/2^*) and control (*Mff^+/+^*; *HD^R6/2^*; *Mff^gt/gt^*) animals (Fig 1A).

**Generation of experimental cohorts and their survival.**
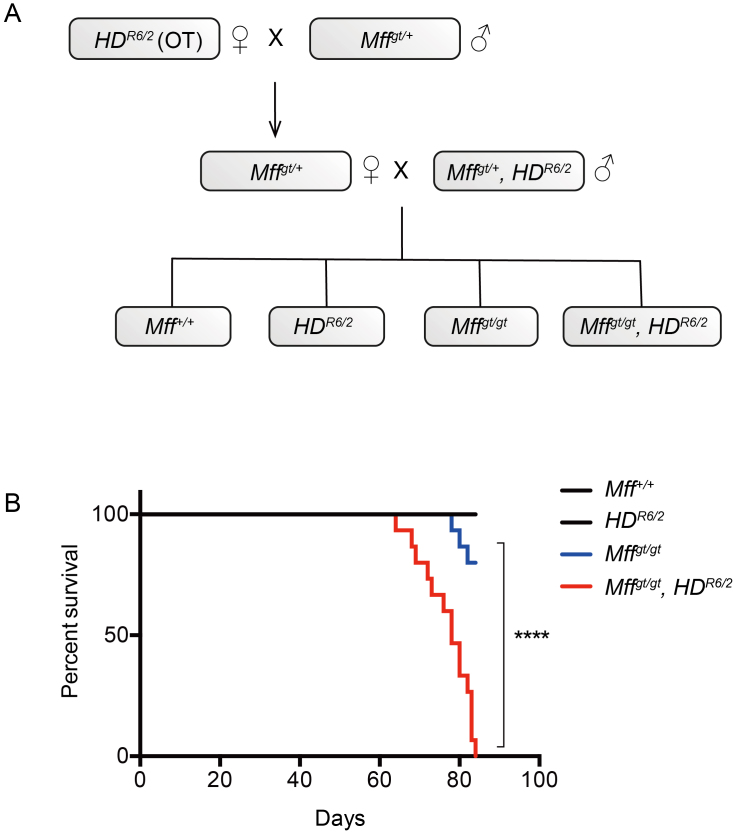
(A) Ovarian transplanted (OT) *HD^R6/2^* females were mated to *Mff^gt/+^* males to generate *Mff*^*gt/+*^, *HD*^*R6/2*^ males. *Mff^gt/+^*, *HD^R6/2^*males were then mated to *Mff*^*gt/+*^ females to generate control (*Mff*^*+/+*^; *Mff^+/+^*, *HD^R6/2^*; *Mff^gt/gt^*) and experimental animals (*Mff^gt/gt^*, *HD^R6/2^*). (B) Kaplan-Meier survival curve of mice of the indicated cohorts. *n* = 15 for all groups. The *p* value represents the log-rank comparison of survival between the *HD^R6/2^* and *Mff^gt/gt,^* *HD*^*R6/2*^ mice. Abbreviations: ****, *p* < 0.0001. The average CAG repeat sizes for the *HD*^*R6/2*^ and *Mff*^gt/gt^, *HD^R6/2^* cohorts were 127.6 and 126.8, respectively.

To determine whether removal of Mff altered the life span in *HD^R6/2^* mice, we evaluated longevity and found that *Mff^gt/gt^*, *HD^R6/2^* mice began dying several weeks earlier than *HD^R6/2^* mice and lived to only ~12 weeks (Fig 1B). In contrast to wildtype mice, *HD^R6/2^* mice show moderate weight loss between weeks 6-11 (Fig. 2A). The weights of *Mff^gt/gt^* mice also lag behind wildtype mice, consistent with our previous results^15^. Interestingly, *Mff^gt/gt^, HD^R6/2^* mice showed a more severe weight loss than either of these mutant mice (Fig 2A; p=0.002). Furthermore, *Mff^gt/gt^*, *HD^R6/2^* mice exhibited markedly higher clasping behavior from 8 to 11 weeks, compared to *HD^R6/2^* mice (Fig 2B). *Mff^gt/gt^*, *HD^R6/2^* mice were significantly weaker than either *Mff^gt/gt^* or *HD^R6/2^*mice in forelimb grip force (Fig 2C). *Mff^gt/gt^*, *HD^R6/2^* mice also showed less spontaneous activity than *HD^R6/2^* mice when allowed to explore an open field chamber, although the result did not reach statistical significance (Fig 2D). Taken together, these findings demonstrate that the Mff knockout exacerbates the behavioral and neurological phenotypes of *HD^R6/2^* mice.

**Loss of Mff exacerbates HD neurological phenotypes.**
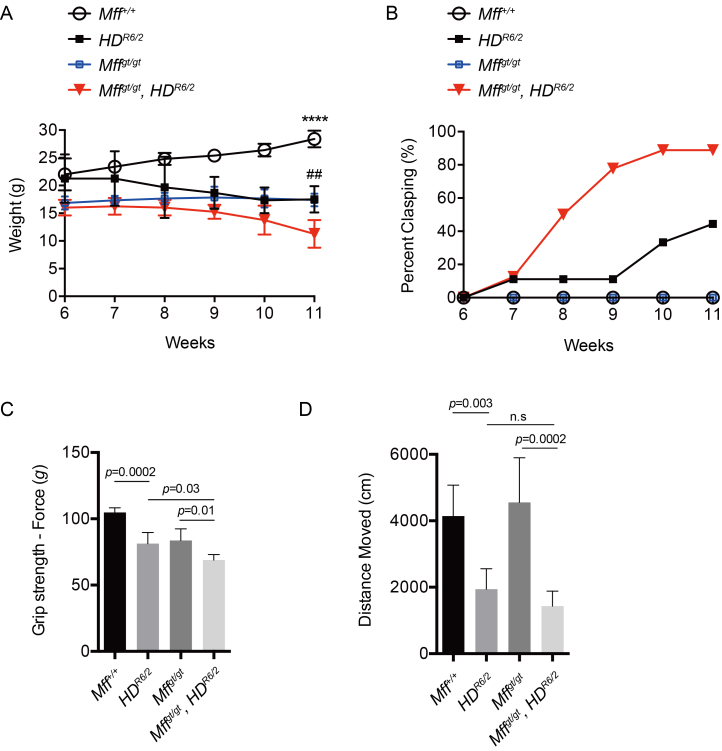
(A) Body weight in male mice was measured at 6-11 weeks of age. *n* = 7 for *Mff^+/+^*; *n* = 4 for *HD^R6/2^*; *n* = 7 for *Mff^gt/gt^*; *n*=4 for *Mff^gt/gt^*, *HD^R6/2^*. (B) Clasping behavior was evaluated upon tail suspension at 6-11 weeks of age. *n* = 9 for all groups. (C) Forelimb grip strength in male mice was examined by a force gauge machine at 11 weeks of age. *n* = 7 for *Mff^+/+^*; *n* = 4 for *HD^R6/2^*; *n* = 7 for *Mff^gt/gt^*; *n*=4 for *Mff^gt/gt^*, *HD^R6/2^*. (D) Spontaneous activity by evaluated by recording the total travelled distance during an open field test at 11 weeks of age. *n* = 9 for all groups. Error bars represent the mean s.e.m. Abbreviations: n.s., not significant; ****, *p* < 0.0001; ##, *p* < 0.01 versus *Mff^gt/gt^*, *HD^R6/2^*. For (A) and (C), the *HD^R6/2^* and *Mff^gt/gt^*, *HD^R6/2^* mice had average CAG repeat sizes of 127.5 and 127.5, respectively; for (B) and (D), 127.3 and 126.0, respectively.

**Modulation of Mff does not alter mutant Htt aggregation in *HD^R6/2^* mice**

To examine the impact of Mff modulation on mutant huntingtin aggregation, we isolated the striatum of each mice and performed immunoblot analysis. In *HD^R6/2^* mice, the detergent-insoluble fraction of the striatum showed high accumulation of mutant Htt aggregates. The levels were unchanged in *Mff^gt/gt^*, *HD^R6/2^* mice (Fig 3A). Using the EM48 antibody to visualize Htt aggregates in striatal sections, we found that the number of Htt inclusions in *HD^R6/2^* mice was unchanged by removal of Mff (Fig 3B). Thus, even though loss of Mff increases the severity of the neurological phenotype in the *HD^R6/2^* model, immunohistochemical and biochemical assays indicate that it does not affect deposition of mutant Htt aggregates.

**Mutant Htt accumulation in the striatum.**
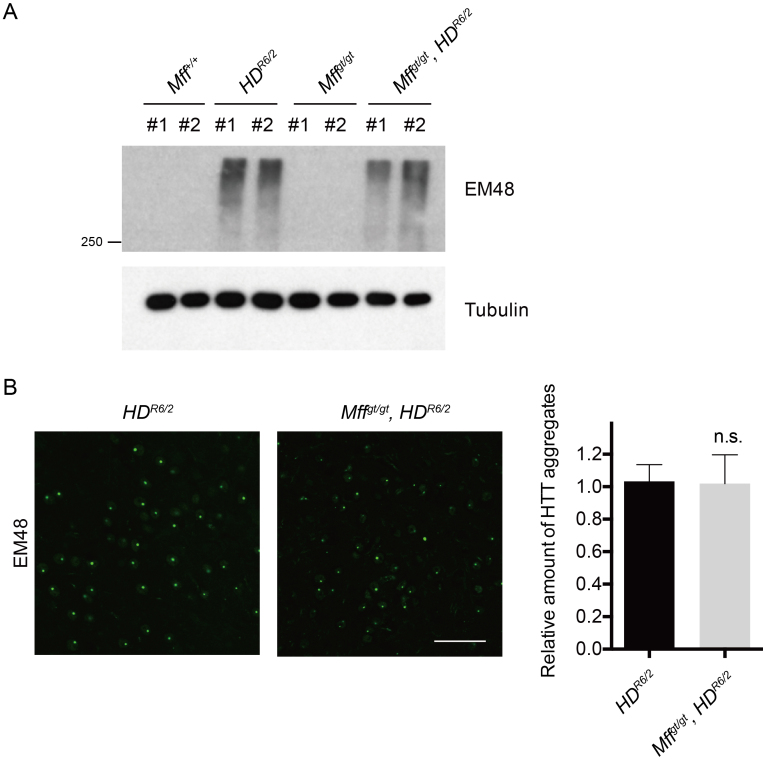
(A) Western blot analysis of insoluble mutant Htt aggregates in striatal brain lysates. Two samples are shown for each genotype. Tubulin was used as a loading control. (B) Immunostaining of mutant Htt aggregates. Coronal brain sections were stained with the EM48 antibody to detect Htt aggregates. Scale bar = 40 μm. (C) Quantification of EM48-positive area (n = 4 per group). Error bars represent the mean s.e.m. Abbreviations: n.s., not significant. For Figures 3-5, the average CAG repeat sizes for the HDR6/2 and Mffgt/gt, HDR6/2 mice were 127.25 and 126.0, respectively.

**Loss of Mff increases neuronal loss and inflammation in *HD^R6/2^* mice**

Previous studies showed that HD mice exhibited extensive neuronal loss in the striatum area^23^^,^^24^. To test whether Mff influences progressive neuronal loss, we performed immunohistochemistry of brain sections among each group with an antibody against NeuN, a neuronal marker protein. Quantitative analysis revealed that *HD^R6/2^* mice showed a decreased number of NeuN-positive neurons relative to wildtype mice, in agreement with previous studies [23, 24] (Fig 4A). Furthermore, *Mff^gt/gt^*, *HD^R6/2^* mice contained markedly fewer (~25%) NeuN-positive neurons compared to *HD^R6/2^* mice. Subsequent examinations with Nissl staining showed similar results (Fig 4B).

**Loss of Mff exacerbates neuronal loss:**
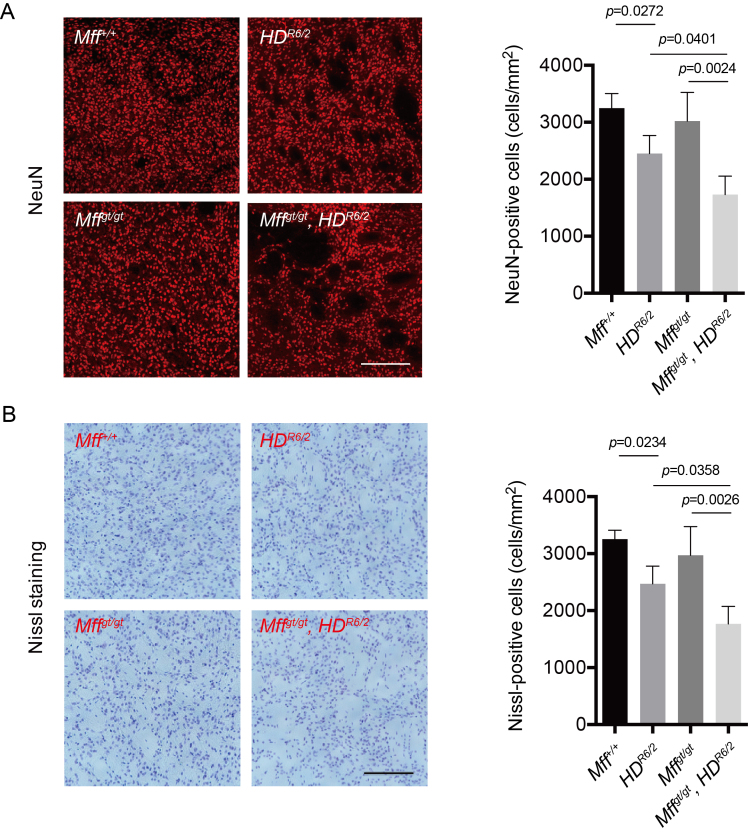
(A) Immunohistochemical staining for NeuN, a neuronal marker (left). Scale bar = 200 μm. Bar graph shows quantification of NeuN-positive cell number (right). n = 4 per group. (B) Representative images of Nissl stained neurons (left). Scale bar = 200 μm. Bar graph shows quantification of Nissl-positive cell number. n = 4 per group. Error bars represent the mean s.e.m.

Accumulation of mutant Htt has been suggested to cause neuroinflammation that potentially promotes neurotoxicity in HD^25^^,^^26^^,^^27^. Neuroinflammation manifests as elevated astrocyte and microglia activation^28^. To measure the neuroinflammatory response, we performed immunostaining with GFAP (glial fibrillary acidic protein), an astrocyte marker (Fig 5A), and Iba-1 (ionized calcium binding adaptor molecule-1), a microglia marker (Fig 5B). Interestingly, we found significantly elevated GFAP and Iba-1 immunoreactivity in *Mff^gt/gt^*, *HD^R6/2^*mice compared to *HD^R6/2^* mice. These results suggest that Mff depletion promotes loss of neurons and an elevated neuroinflammatory response in *HD^R6/2^* mice.

**Loss of Mff exacerbates astrogliosis and inflammation:**
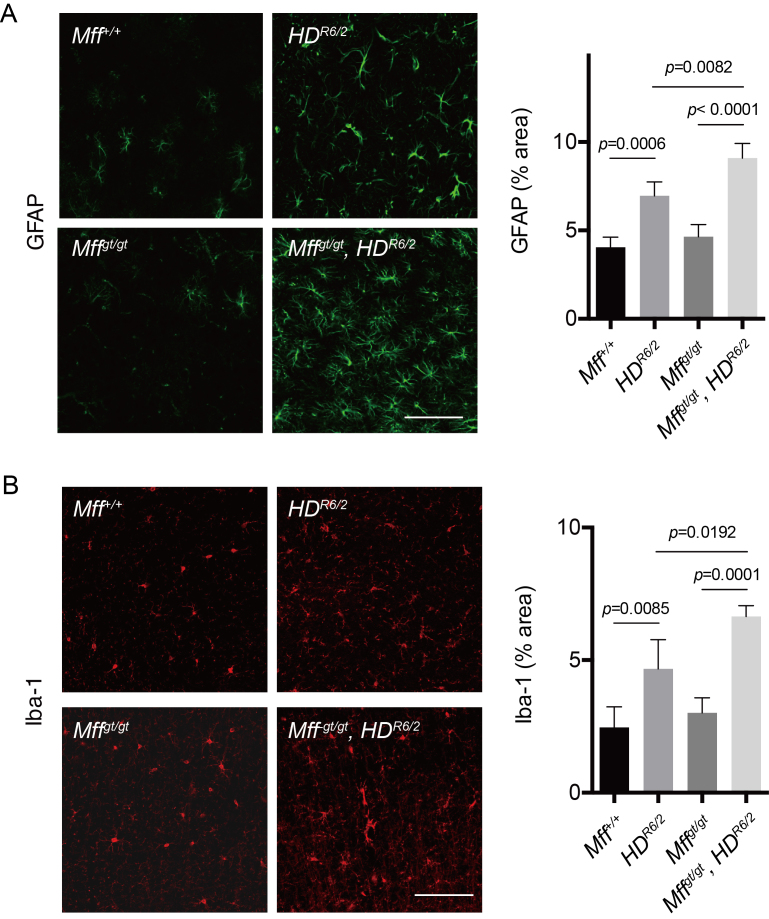
Loss of Mff exacerbates astrogliosis and inflammation. (A) Immunohistochemical staining for GFAP, an astrocyte marker (left). Scale bar = 100 μm. Bar graph shows quantification of GFAP-positive area (right). *n* = 4 per group. (B) Immunohistochemical label for Iba-1, a marker for TFNγ-induced marker of activated microglia (left). Scale bar = 100 μm. Bar graph shows quantification of Iba-1-positive area (right). *n* = 4 per group. Error bars represent the mean s.e.m.

## DISCUSSION

HD cells have been shown to have aberrantly increased mitochondrial fragmentation, an effect attributed to increased activation of Drp1 and fission^10^^,^^11^. Given that peptide-based inhibition of Drp1 has been reported to ameliorate the neurological symptoms and mortality of *HD^R6/2^* mice^21^, we wondered whether a similar effect would be found with removal of Mff. Mff is a major receptor for Drp1, and embryonic fibroblasts from our *Mff^gt/gt^*mice have substantially reduced recruitment of Drp1 and fission activity^16^^,^^17^. Its role in mitochondrial fission has been shown in a variety of cultured cells from Drosophila, human, and mouse, and it is expressed in the mammalian brain^30^. However, we found no evidence that removal of Mff could improve the phenotype of *HD^R6/2^* mice. We found instead that loss of Mff resulted in more severe neurological symptoms and earlier lethality. Loss of Mff did not increase the levels of aggregated Htt, but did increase loss of neurons, astrogliosis, and neuroinflammation.

P110 was designed to block a putative interaction between Drp1 and Fis1^22^. The function of Fis1 in Drp1 recruitment to mitochondria remains unclear, due to the observation that cells lacking Fis1 show little or no defect in Drp1 recruitment or mitochondrial fission^16^^,^^17^. It remains possible that Fis1 does play a role in mitochondrial fission in specialized cell types or under particular cellular stress conditions. P110 has also been shown bind recombinant Drp1 directly and inhibit its GTP hydrolysis activity^22^. More work will be required to understand the mechanism through which P110 affects the phenotype of *HD^R6/2^* mice.

Our results indicate that loss of Mff aggravates the neurological symptoms of *HD^R6/2^*mice. Therefore, although there is evidence that inhibition of Drp1 function can improve the phenotype of *HD^R6/2^* mice^21^, Mff seems to not be the relevant Drp1 receptor for this effect. The P110 results suggest a role for Fis1. MiD49 and MiD51 also remain possibilities. It is currently unclear why there are potentially four Drp1 receptors, with each playing a role in mitochondrial fission^17^^,^^31^^,^^32^. This diversity of Drp1 receptors may allow regulation of Drp1 function to be tailored to the cellular state. For example, Mff, MiD49, and MiD51 have different effects on Drp1 function upon recruitment. Unlike Mff, MiD49 and MiD51 have inhibitory effects on Drp1 function^17^^,^^31^ , and MiD51 has been shown to inhibit the GTP hydrolysis activity of Drp1 [30]. Additional stimuli are presumably necessary to activate Drp1 once it has been recruited by MiD49 or MiD51. MiD49 and MiD51 also appear to play stronger roles in mediating mitochondrial fission during apoptosis compared to Mff^32^.

Our results further suggest that Mff is protective in the context of Htt containing an expanded polyglutamine repeat. With Mff is removed, there is increased neuronal cell loss, increased astrogliosis, and increased expression of neuroinflammatory markers. These detrimental effects may arise because loss of Mff upsets the delicate balance between mitochondrial fusion and fission, and as a result neurons are less able to cope with Htt aggregates. Our previous mouse studies suggest that an appropriate balance between these opposing processes is critical for mitochondrial health. Multiple setpoints for fusion versus fission are compatible with mitochondrial function, but the levels have to been carefully balanced^15^. *Mff*^*gt/gt*^ mice show reduced respiratory chain function, as shown in cardiomyocytes, and this dysfunction is associated with reduced mitochondrial density and aberrant mitophagy^17^. If these cellular defects extend to neurons, they may help to explain the worsening of the *HD^R6/2^* phenotype.

## CONCLUSIONS

Although inhibition of mitochondrial fission has been proposed as a therapeutic approach for HD, we find that removal of Mff, a mitochondrial fission factor, exacerbates the neurological phenotypes of *HD^R6/2^* mice.Therefore, our results indicate that a deeper understanding of mitochondrial dynamics in HD is required before mitochondrial fission can be considered a therapeutic avenue for HD.

## Data Availability Statement

The raw data for graphs in Figures 1-5 are available at https://figshare.com/s/584ca97ed838e5de3bde, with DOI: 10.6084/m9.figshare.6052007.

## Competing Interests Statement

The authors have declared that no competing interests exist.

## Funding

This work was funded by grant A-11059 from the CHDI Foundation (https://chdifoundation.org). The funder had no role in study design, data collection and analysis, decision to publish, or preparation of the manuscript.
